# Heat Transfer Capability of (Ethylene Glycol + Water)-Based Nanofluids Containing Graphene Nanoplatelets: Design and Thermophysical Profile

**DOI:** 10.1186/s11671-016-1806-x

**Published:** 2017-01-19

**Authors:** D. Cabaleiro, L. Colla, S. Barison, L. Lugo, L. Fedele, S. Bobbo

**Affiliations:** 1 Departamento de Física Aplicada, Facultade de Ciencias, Universidade de Vigo, E-36310 Vigo, Spain; 2 Istituto per le Tecnologie della Costruzione, Consiglio Nazionale delle Ricerche, Padova, Italy; 3 Istituto di Chimica della Materia Condensata e di Tecnologie per l’Energia, Consiglio Nazionale delle Ricerche, Padova, Italy

**Keywords:** Graphene nanoplatelets, Ethylene glycol + water, Nanofluid, Thermal conductivity, Dynamic viscosity, Volumetric behavior, Heat transfer capability, Pumping power

## Abstract

This research aims at studying the stability and thermophysical properties of nanofluids designed as dispersions of sulfonic acid-functionalized graphene nanoplatelets in an (ethylene glycol + water) mixture at (10:90)% mass ratio. Nanofluid preparation conditions were defined through a stability analysis based on zeta potential and dynamic light scattering (DLS) measurements. Thermal conductivity, dynamic viscosity, and density were experimentally measured in the temperature range from 283.15 to 343.15 K and nanoparticle mass concentrations of up to 0.50% by using a transient plate source, a rotational rheometer, and a vibrating-tube technique, respectively. Thermal conductivity enhancements reach up to 5% without a clear effect of temperature while rheological tests evidence a Newtonian behavior of the studied nanofluids. Different equations such as the Nan, Vogel-Fulcher-Tamman (VFT), or Maron-Pierce (MP) models were utilized to describe the temperature or nanoparticle concentration dependences of thermal conductivity and viscosity. Finally, different figures of merit based on the experimental values of thermophysical properties were also used to compare the heat transfer capability and pumping power between nanofluids and base fluid.

## Background

As energy transport processes are integrated in most industrial areas, the development of more efficient and compact heat transfer equipment has focused a lot of attention during the last decades. Once different approaches such as material modifications, the use of extended surfaces, or the optimization of process parameters have been extensively exploited, many research activities are focused now on improving the weak heat-transfer abilities of typical conventional fluids such as water (W), ethylene glycol (EG), or engine oils [1, 2]. In this sense, the enhancement of thermal transport properties of heat transfer fluids by dispersing high-thermal conductive nanomaterials, also known as nanofluids, has become a prominent research avenue [3, 4].

A wide variety of nanoadditives, including metallic, organic, and non-organic materials, has been utilized to engineer nanofluids. Among them, carbon nanostructures are those which seem to exhibit the highest potential [5]. Within the family of graphite carbon allotropes, the remarkable mechanical, structural, thermal, and electrical properties of graphene [6–8] have raised great interest since this material was discovered by Novoselov et al. [9] in 2004. Graphene structure is ideally envisaged as a single-atom-thick sheet of hexagonally arranged, sp^2^-bonded carbon atoms tightly packed into a honeycomb lattice [10, 11]. This two-dimensional material is available commercially in the form of several-layer stacks (usually more than 10 layers) which are known as graphene nanoplatelets or nanosheets (GnPs).

GnPs combine the advantages of both single-layer graphene and highly ordered graphitic carbon [12, 13]. Thus, the thermal conductivity of graphene nanoplatelets has been reported to be much higher than the value presented by other carbon allotropes like multi-wall nanotubes (MWNT), single-wall nanotubes (SWNT), or diamond [8, 14]. In addition, graphene nanosheets possess larger surfaces areas compared to nanotubes or other nanoparticles, allowing bigger contact areas/interfaces with the base fluid. This superior contact could lead to a reduction of Kapitza resistance at the graphene-fluid interface and, consequently, help to improve the effective thermal conductivity of the nanofluid [15]. Another upside of graphene nanomaterials is the relatively easy and cost-effective production at a large scale [13, 16, 17]. Hence, graphene appears to meet all the conditions to develop new nanofluids with improved thermal properties. Nevertheless, unfortunately, graphene is hydrophobic and consequently it cannot be dispersed in water or some polar organic solvents for a long time without agglomerating [18, 19]. In order to increase the dispersibility, the interactions between graphene nanostructures and aqueous/organic solvents can be improved by means of non-covalent (using surfactants) or covalent (adding hydrophobic or hydrophilic groups on the high energy features such as edges of graphene nanosheets) functionalizations [16].

In water- and ethylene glycol-based nanofluids, the surfactants predominating in the literature for non-covalent options are sodium dodecyl benzene sulphonate (SDBS) [16, 20–22], polyvinyl alcohol (PVA) [23], and gum Arabic [14, 22], while the oxidation following the original version [24, 25] or modifications [26] of Hummers method are the most common with covalent functionalizations. The main downside of using non-covalent methods is that the addition of a surfactant considerably increases the viscosity of the nanofluid, leading to higher pressure drops in thermal equipment. As an example, Amiri et al. [16] found that the viscosity enhancement of non-covalent graphene/water nanofluids synthesized using SDBS doubles the values of covalent functionalized nanofluids at the same nanoparticle concentration. Regarding covalent functionalizations, although Paredes et al. [27] reported that good dispersions of graphene oxide (GO) can be obtained not only in water but also in other organic solvents such as ethylene glycol, acid treatment can lead to the formation of defect sites within the systematically arranged conjugated graphene structure of the nanosheets [28–30]. As these defects could reduce the thermal conductivity of graphene by an order of magnitude or more below its intrinsic value, some reaction conditions such as acid content, temperature, or time of exposure must be optimized in order to minimize overoxidation and subsequent defect formation [11]. Another peculiar property of graphene oxide is the high natural acidity of its aqueous solutions due to the generation of hydrogen cations resulting from GO-water interactions [31, 32]. Thus, a special attention must be paid to pH control in order to avoid possible damages to metallic elements of the installation [33].

During the last decades, thermal conductivity (*k*) of nanofluids has received a lot of attention due to the high influence of this property on the heat transfer performance of thermal facilities [34, 35]. However, other thermophysical properties, such as dynamic viscosity (*η*), density (*ρ*), or even specific heat capacity (*c*_*p*_), should also be taken into account in order to assess whether the replacement of the conventional fluid with the new nanofluid would be really beneficial, as well as to make technical calculations of thermal facilities. Dynamic viscosity or the rheological characteristics in general have a critical effect on the type of flow and consequently on the heat transfer and the necessary pumping power, for example [36]. Although the complex behavior of nanofluids precludes a generalization of their thermophysical properties, the addition of nanoparticles usually leads to higher thermal conductivities, viscosities, and densities as well as to lower specific heat capacities. The improvement of some properties and the worsening of others hinders the choice of the fluid with the best features, and so it is necessary to use figures of merit (FoMs) such as the Mouromtseff number (Mo) [37] in order to select the fluid with better heat transfer capabilities.

Over the last years, an important number of studies about graphene nanofluids have been performed using water [1, 3, 13, 38–41] or ethylene glycol [12, 20, 31, 39, 40] as base fluids. However, many industrial facilities do not utilize these pure compounds as heat transfer media but their mixtures in order to combine the advantages of both compounds, such as the better thermal conductivity of water or the lower freezing points of glycols. To our knowledge, only Kole and Dey [15], Amiri et al. [42], and Ijam et al. [43] have analyzed the thermophysical properties of graphene nanofluids based on (ethylene glycol + water) mixtures. Kole and Dey [15] studied the thermal conductivity and rheological behavior of functionalized hydrogen exfoliated graphene in EG + W (70:30 vol.%) at nanoparticle concentrations between 0.041 and 0.395 vol.%. They found that both properties increase with the addition of graphene, maximum increases reaching 15% for thermal conductivity and 100% for dynamic viscosity. Amiri et al. [42] used a mixture of EG + W (60:40 vol.%) as base fluid to prepare nanofluids at graphene mass concentrations from 0.01 to 0.2%. Thermal conductivity, density, and viscosity were reported to rise up to (65, 0.6, and 4.9)%, respectively, for the highest concentration. Contrarily, specific heat capacity decreases up to 5%. Ijam et al. [43] studied dispersions of graphene oxide nanosheets in EG + W (40:60 vol.%) finding thermal conductivity and viscosity increases at the highest concentration (0.10 wt.%) of up to 10.5 and 35%, respectively.

In this work, sulfonic acid-functionalized graphene nanoplatelets were dispersed in a (ethylene glycol + water) mixture at (10:90)% mass ratio to prepare nanofluids at (0.10, 0.25, and 0.50)% nanoparticle mass fractions, which correspond to 0.00045, 0.00112, and 0.00225 nanoparticle volume fractions, respectively. Volume fractions were calculated by using a density value of 2.25 g cm^−3^ [28] for graphene oxide and the experimental densities obtained in this work for base fluid. Preparation conditions such as sonication time or pH value were optimized by studying the zeta potential and variation of the average nanoparticle size with time. Thermal conductivity, dynamic viscosity, and density of nanofluids and base fluid were obtained experimentally, and the influences of temperature and nanoparticle concentration on these three properties were analyzed. Finally, the modifications of heat transfer performance and pumping power were assessed from the thermophysical properties here obtained by means of different figures of merit.

## Methods

### Materials

Sulfonic acid-functionalized graphene oxide nanoplatelets (GOnPs) were provided by NanoInnova Technologies S.L. (Madrid, Spain). The base fluid consists of a (ethylene glycol + water) mixture at (90:10)% mass ratio. Ethylene glycol was purchased from Sigma-Aldrich with a mass purity of 99.5% while Milli-Q Grade water was produced with a resistivity of 18.2 MΩ cm at 298 K by means of a Millipore system (Billerica, MA, USA). An ammonium hydroxide solution (30–33% NH_3_ in water) from Sigma-Aldrich was used for the modification of the pH value. A Sartorius analytical balance with an uncertainty of 0.0001 g was utilized to weigh the reagents.

### Powder Characterization, Nanofluid Preparation, and Stability Analysis

**Fig. 1:**
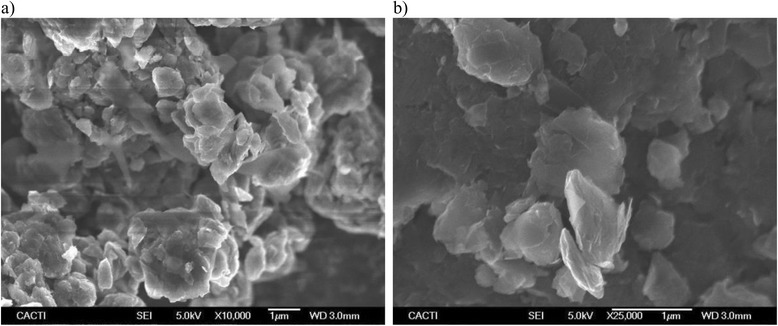
Morphology of GOnP powder. SEM images of GOnP particles at two magnifications: **a** ×10,000 and **b** ×25,000

The morphology of the dry powder was studied through scanning electron microscopy analyses conducted on a JEOL JSM-6700F field emission gun-SEM (JEOL, Tokyo, Japan) working at an accelerator voltage of 20 kV in backscattering electron image (Yttrium Aluminum Garnet type detector). SEM samples were prepared by depositing a drop of a GOnP dispersion in analytical grade methanol on the top of a silica support and drying it under atmospheric conditions. A typical SEM image of GOnP additive is presented in Fig. 1. GO particles exhibit a plate-like shape of up to some micrometers, rough surfaced with tiny wrinkles over the whole surface and mountainous peaks similar to those presented by Geng et al. [44]. The edges are not flat and smooth but rounded, which may be due to the synthesis route as also pointed out in [44]. A further characterization of the studied GOnP nanoplatelets can be found in Agromayor et al. [45].

Nanofluids were prepared through a two-step process. Thus, a predetermined amount of graphene nanopowder was first added to the mass of liquid necessary to obtain the desired nanoparticle concentration and then stirred for 120 min. Afterwards, samples were sonicated by means of a CP104 Ultrasonic Bath (CEIA, Italy) working with a maximum sonication power of 200 W and a sonication frequency of 40 kHz. As it was pointed out, one of the disadvantages of graphene oxide nanoplatelets is the high acidity of its aqueous solutions due to the presence of surface acidic groups [31, 32]. Thus, in our study, pH values between 2.2 and 2.8 were measured for the nanofluids once the nanomaterial was added. In order to modify the pH conditions, the pH value of dispersions was raised by adding ammonium hydroxide. pH measurements were performed with a PHM 210 standard pH meter (Radiometer Analytical S.A., France) with a pH electrode code 5208 (Crison Instruments SA, Spain). With the aim of optimizing nanofluid design, the influences of the pH value and sonication procedure on nanofluid stability were evaluated through zeta potential and size measurements by using a Zetasizer Nano (Malvern, UK) [46].

**Fig. 2:**
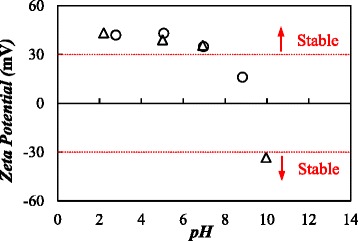
Zeta potential of GOnP/(EG + W) nanofluids. Zeta potential as a function of pH value at different mass concentrations: *circle*, 0.10%; and *triangle*, 0.25%

Firstly, zeta potential was studied for 0.10 and 0.25% nanoparticle mass concentrations in the pH range from 2.2 to 10. No zeta potential measurements were performed for the highest mass concentration since the intensity detected by the device was outside the optimum working range. Figure 2 shows the pH influence on the zeta potential of graphene nanofluids. As can be observed, zeta potential remains constant around 40–43 mV for pH values up to 5 and then decreases as pH value rises. In order to reach a compromise between high zeta potentials to ensure strong electrical repulsion charges around the particles and appropriate pH values to avoid corrosion issues, a pH = 5 was selected to prepare the nanofluids.

**Fig. 3:**
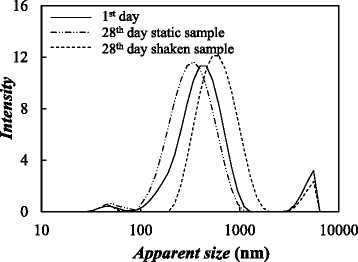
Apparent size distributions of GOnP/(EG + W) nanofluids. Apparent size distributions of the 0.25% mass concentration: *solid line*, 1st day; *dash-dot line*, static sample at the 28th day; and *dashed line*, shaken sample at the 28th day

The nanoparticle size distribution of the nanofluids was determined by measuring the random changes in the intensity of light scattered from the dispersions at 298.15 K and with a scattering angle of 173°. This technique was used to select the optimum sonication time determining “apparent” size measurements for nanofluids prepared with 0.10 wt.% GOnP concentration and sonication times from 0 to 300 min. It should be pointed out that dynamic light scattering (DLS) measurements are based on the assumption that particles are spherical while the studied nanoadditives are sheet-like shaped. The DLS results indicate that the graphene nanoplatelets of the fresh nanofluids exhibit a trimodal distribution with apparent sizes from some nanometers to 4 μm. This polydispersity of graphene nanofluids was previously reported in literature [24, 47]. Width and medium value of the different peaks of the size distribution reduce as sonication time increases. However, this reduction mainly occurs at times between 0 and 240 min. while only slight variations were found between samples prepared at 240 and 300 min. Thus, a sonication time of 300 min was selected to prepare the nanofluids at the three studied nanoadditive concentrations. Subsequently, the evolution of nanofluid stability with the time elapsed after preparation was studied following the procedure defined by Fedele et al. [46]. Thus, two samples of each nanoparticle concentration were put in two different cuvettes and their apparent sizes were studied by DLS for a month. The sample of the first cuvette was measured almost every day without shaking the fluid (in static conditions) in order to evaluate the changes in the size distribution due to natural sedimentation, while the second sample was measured after being manually shaken for some seconds to measure apparent size distribution after mechanically recovering settled particles. The apparent size distributions of the 0.10 wt.% graphene concentration measured just after preparation and the 28th day are plotted in Fig. 3. As it can be observed, in the case of the static samples, the main peak slightly moves to the left and the 4000-nm peak disappears while the 50-nm and 400-nm peaks move to the right for the shaken sample. This indicates a partial agglomeration of nanoadditives and sedimentation (especially of the largest nanoplatelets) under static conditions.

### Experimental Methods

Thermal conductivities were determined at temperatures ranging from 293 to 343 K by means of a TPS 2500 S Hot Disk Thermal Analyzer® (Hot Disk AB, Sweden), which is appropriate to perform measurements of different materials in the range from 0.005 to 500 W m^−1^ K. This instrument was used together with a 7877 sensor based on the transient plate source (TPS) and consisting of a double spiral of 2-mm radius made of thin nickel wire. The probe, which works as both heat source and temperature sensor, was vertically immersed in the sample so that heat could freely diffuse in all directions. In order to ensure a uniform initial temperature and remove thermal gradients, the box which contains both sample and probe was placed in a thermostatic bath and measurements of thermal conductivity were performed at least with a delay of 15 min between them. Tests were carried out using low thermal powers, 40–55 mW, and a short power input time, 4 s, in order to minimize convection effects. With the aim of checking the measuring procedure in the studied temperature range, the thermal conductivity of bidistilled water was measured and the results were compared with the NIST database [38]. Maximum deviations between the here reported thermal conductivities and literature data [38] are lower than 1.5%, well within the 5% accuracy reported by the manufacturer. Additional information about the experimental device and measuring procedure can be found in Fedele et al. [48].

Rheological behavior was studied by using an AR-G2 magnetic bearing rotational rheometer (TA Instruments, New Castle, USA) with a cone-plate geometry of 1° cone angle and 40-mm diameter. This device allows controlling torques from 0.1 μN m to 200 mN m and normal forces between 0.005 and 50 N. The sample temperature was regulated in the range between 293 and 343 K by using a Peltier plate and an upper heated plate (UHP). Before tests, the rheometer was carefully calibrated at each temperature as further described by Bobbo et al. [49]. The geometry was imposed to a gap of 30 μm and an amount of 0.34 cm^3^ was considered optimal for the experiments. Flow curve tests were carried out at constant temperature and shear rates ranging from 80 to 1200 s^−1^. In order to assess measurement accuracy, water (a fluid of well-known viscosity) was studied in the entire temperature range and the values were compared with those of the NIST database [38]. Average Absolute Deviations, AADs%, between both data sets are less than 2% which is well within the uncertainty reported by the manufacturer for viscosity measurements, i.e., 5%.

Densities were experimentally obtained with an Anton Paar DMA 4500 vibrating U-tube densimeter, which relates sample density and oscillation period. Temperature was controlled with a resolution of 0.01 K in the range from 293 to 343 K by means of a solid-state thermostat. Calibration was performed using water and air, and uncertainty was estimated to be 5 × 10^−4^ g cm^−3^ [50].

## Results and Discussion

### Thermal Conductivity

**Fig. 4:**
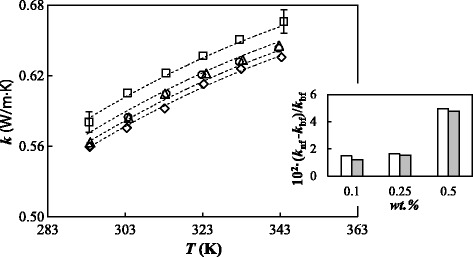
Thermal conductivity of GOnP/(EG + W) nanofluid set. Temperature dependence at different mass concentrations: *diamond*, base fluid 0%; *circle*, 0.10%; *triangle*, 0.25%; and *square*, 0.50%. The *dashed lines* represent the fitted Nan model [55], Eqs. (2–3). The *inset* shows the thermal conductivity enhancements of the nanofluids in relation to the base fluid, 10^2^ (*k*_nf_ − *k*_bf_)/*k*_bf_, at 303.15 K (*white bars*) and 343.15 K (*gray bars*)

The thermal conductivity of the base fluid and the three designed nanofluids was measured at ambient pressure in the temperature range from 293 to 344 K with steps of 10 K. The temperature dependence of experimental values is depicted in Fig. 4. To our knowledge, only Melinder [33] reported the thermal conductivities of (ethylene glycol + water) mixture at (10:90)% mass concentration. In addition, Sun et al. [51] and Bohne et al. [52] studied different concentrations of (ethylene glycol + water) system and proposed thermal conductivity correlations as a function of composition and temperature. The obtained values for the base fluid were compared with these existing literature data. AADs% of (2.6, 1.9, and 4.0)% between our experimental values and those reported or calculated by using the equations proposed by Melinder [33], Sun et al. [51], and Bohne et al. [52] were obtained, respectively. For each fluid, the temperature dependence was described by using a second-order polynomial:

1 $$ k(T)={a}_0+{a}_1\cdotp T+{a}_2\cdotp {T}^2 $$

where the *a*_i_ adjustable coefficients were obtained with AADs% lower than 0.3% by using a least-square fitting and are gathered in Table 1. Thermal conductivity increases with temperature up to 14.7% throughout the entire studied range. It also increases up to 5% with the addition of nanoparticles, and the enhancements have no temperature dependence as showed in the inset of Fig. 4. The reported temperature-independent thermal conductivity enhancements are in agreement with those found by Yu et al. [53] and Hadadian et al. [31] for GOnP/EG nanofluids or by Kole and Dey [15] for hydrogen exfoliated graphene-(EG + W) nanofluids.

**Table 1:** Adjustable parameters, AADs%, and standard deviations, *σ*, of the different correlations presented for GOnP/(EG + W) nanofluids

	Base fluid (0 wt.%)	0.10 wt.%	0.25 wt.%	0.50 wt.%
Second-order polynomial fitting, Eq. (1)				
*a*_0_/(W m^−1^ K^−1^)	−0.86582	−1.8363	−1.3787	−1.0881
10^3^ *a*_1_/(W m^−1^ K^−2^)	7.6323	13.706	10.831	9.1277
10^6^ *a*_2_/(W m^−1^ K^−3^)	−9.4810	−18.883	−14.374	−11.716
AADs%	0.3	0.1	0.1	0.2
*σ*/(W m^−1^ K^−1^)	0.002	0.001	0.001	0.002
Vogel–Fulcher–Tammann (VFT) model, Eq. (4)				
*η*_0_/(mPa s)	0.0470	0.0488	0.0504	0.0544
*D*/K	2.41	2.42	2.42	2.45
*T*_0_/K	169.50	168.82	168.88	166.72
AADs%	1.3%	1.6%	1.6%	1.1%
*σ*/(mPa s)	0.017	0.018	0.018	0.016
Second-order polynomial fitting, Eq. (6)				
*b*_0_/(g cm^−3^)	0.80296	0.80933	0.82096	0.82813
10^3^ *b*_1_/(g cm^−3^ K^−1^)	1.6936	1.6583	1.5845	1.5493
10^6^ *b*_2_/(g cm^−3^ K^−2^)	−3.3571	−3.3036	−3.1786	−3.1250
AADs%	0.003	0.007	0.010	0.003
10^4^ *σ*/(g cm^−3^)	0.6	1.1	1.5	0.6

*a*_i_ coefficients from the second-order polynomial fitting of thermal conductivity, Eq. (1); *η*_0_, *D*, and *T*_0_ coefficients from Vogel–Fulcher–Tammann (VFT) correlation, Eq. (4); as well as *b*_i_ coefficients from the second-order polynomial fitting of density, Eq. (6), for GOnPs/(EG + W) nanofluids

During the last decades, several theoretical or semi-empirical equations, based on Maxwell model [54] for spherical and well-dispersed particles, have been developed in order to predict or describe the thermal conductivity of nanofluids. A good agreement was found in literature between experimental data and the values obtained using Nan model [55] for different nanofluids in general [56] and for graphene nanoplatelet dispersions in particular [15, 20, 31, 57, 58]. Nan et al. [55] generalized Maxwell equation including the effects of particle geometry and finite interfacial resistance by the following expression:

2 $$ {k}_{\mathrm{nf}}={k}_{\mathrm{bf}}\cdot \frac{3+\varphi \cdot \left[2\cdot {\beta}_{11}\cdot \left(1-{L}_{11}\right)+{\beta}_{33}\cdot \left(1-{L}_{33}\right)\right]}{3-\varphi \cdot \left(2\cdot {\beta}_{11}\cdot {L}_{11}+\cdot {\beta}_{33}\cdot {L}_{33}\right)} $$

where *L*_ii_ are the geometrical factors which take a value of *L*_11_ = 0 and *L*_33_ = 1 in the case of nanoplatelets [15, 20], *ϕ* is the volumetric fraction of particles, and *β*_ii_ coefficients are defined as:

3 $$ {\beta}_{\mathrm{ii}}=\frac{k_{\mathrm{np}}-{k}_{\mathrm{bf}}}{k_{\mathrm{bf}}+{L}_{\mathrm{ii}}\cdot \left({k}_{\mathrm{np}}-{k}_{\mathrm{bf}}\right)} $$

Based on that model, in-plane thermal conductivity of the nanomaterial, *k*_np_, can be obtained by using a least squares fitting of experimental data of the nanofluids, *k*_nf_, and base fluid, *k*_bf_. Thus, the thermal conductivity obtained by using Eqs. (2–3) for the studied graphene nanoplatelets is 17 W m^−1^ K, which is slightly higher than the values reported by Kole and Dey [15], Yu et al. [20], or Hadadian et al. [31] when studied graphene oxide dispersions in (ethylene glycol + water), ethylene glycol and water, respectively. These differences can be due to the density of defects, size, and/or roughness of graphene oxide, as it was theoretically demonstrated by Nika et al. [59]. The values provided by Nan model are plotted together with the experimental values in Fig. 4, exhibiting a deviation of 0.6%, which is well within the experimental uncertainty.

### Dynamic Viscosity

**Fig. 5:**
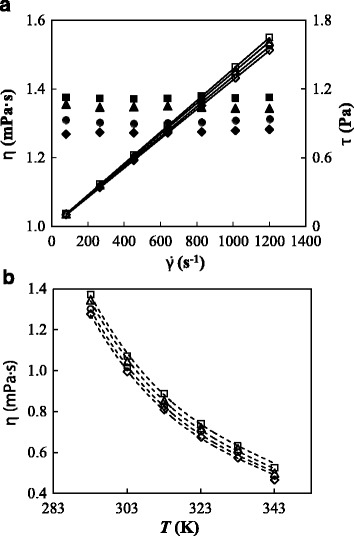
Rheological behavior of GOnP/(EG + W) nanofluid set. **a** Shear rate, $\overset{.}{\gamma }$, vs. dynamic viscosity, *η*, (*filled symbols*) and shear stress, *τ*, (*empty symbols*) at 293.15 K and different mass concentrations: *diamond*, base fluid 0%; *circle*, 0.10%; *triangle*, 0.25%; and *square*, 0.50%. **b** Temperature dependence of dynamic viscosity, *η*, at different mass concentrations: *diamond*, base fluid 0%; *circle*, 0.10%; *triangle*, 0.25%; and *square*, 0.50%. The *dashed lines* represent the fitted VFT model, Eq. (4)

**Fig. 6:**
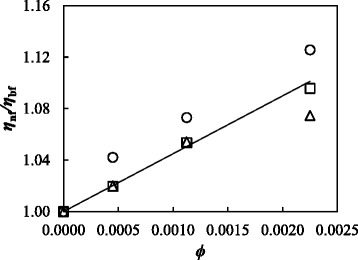
Viscosity modelling. Viscosity ratio, *η*_nf_/*η*_bf_, vs. volume concentration, *ϕ*, at different temperatures: *triangle*, 293.15 K; *square*, 303.15 K; and *circle*, 343.15 K. The *solid line* represents the values provided by Maron-Pierce (MP) relationship, Eq. (5)

The influence of GOnP concentration on the rheological behavior was analyzed at temperatures ranging from 293.15 to 343.15 K with steps of 10 K. Figure 5a shows the flow curves of the base fluid and different studied nanofluids at 293.15 K. Dynamic viscosities obtained for the base fluid were compared with previous literature data [33, 60–62] for the (ethylene glycol + water) mixture at (10:90)% mass ratio. The AADs% between these values range from 1.5 to 3.6%, showing a good agreement. On the other hand, the linear relationship between shear stress and shear rate indicates that both base fluid and nanofluids exhibit a Newtonian behavior over the studied conditions. This Newtonian behavior coincides with the results found by Kamatchi et al. [57] and Mehrali et al. [58] for graphene oxide-water nanofluids at shear rates higher than 80 s^−1^ or by Ma et al. [63] for nanofluids based on dimethyl silicone oil, for example. The temperature dependence of dynamic viscosity measurements is plotted in Fig. 5b. As can be observed, dynamic viscosity decreases considerably with temperature and a modification of Andrade’s equation, which is also known as the three-coefficient Vogel-Fulcher-Tamman (VFT) model, was used to describe this temperature dependence:

4 $$ \ln\ \eta (T)= \ln\ {\eta}_0+\frac{D\cdot {T}_0}{T-{T}_0} $$

where *η*_0_, *D*, and *T*_0_ are the adjustable coefficients. *D* and *T*_0_ parameters are also known as the Angell Strength and Vogel temperature, respectively. The values of these coefficients as well as AADs% and standard deviations between experimental values and data fitted by using this equation are gathered in Table 1. The good description of the temperature dependence, with standard deviations lower than 0.02 mPa s, is also shown in Fig. 5b. The base fluid and studied nanofluids exhibit small values of Angell Strength parameter, which indicates that the fluids present a rapid breakdown of their configurational structure with increasing temperature near and above the glass transition [64, 65]. Figure 6 shows the influence of GOnP concentration on viscosity ratio, i.e., *η*_nf_/*η*_bf_, at several temperatures. As expected, the dynamic viscosity of nanofluids increases with the addition of graphene oxide nanoplatelets. These increases rise with increasing temperature, especially for the 343-K isotherm reaching a maximum increment of 12.6% for the 0.50 wt.% GOnP mass concentration.

The relative viscosity of non-interacting and stationary hard particles in infinitely dilute suspensions can be described by using different equations derived from the pioneering Einstein model [66]. In this sense, Maron and Piece (MP) [67] proposed an equation with the same functional form than that of Krieger-Dougherty relationship [68]. Nevertheless, unlike Krieger-Dougherty model, MP equation does not require the knowledge of intrinsic viscosity, which is not always an easy task when particles are not spherical [69, 70]. The Maron and Piece model [67] has already been successfully utilized to model viscosity enhancements of particles with different aspect rations such as fibers [71, 72] or platelets/flakes [73, 74] and can be written as follows:

5 $$ \frac{\eta_{\mathrm{nf}}}{\eta_{\mathrm{bf}}}={\left(1-\frac{\varphi_a}{\varphi_m}\right)}^{-2} $$

where *ϕ*_a_ is the effective volume fraction of nanoparticles which can be reduced to the volume fraction of nanoparticles in absence of aggregates, while *ϕ*_m_ is the maximum volume packing fraction and can be used as a fitting parameter [70]. In this case, an ADD% of 1.3% and a maximum deviation of 2.8% between experimental and correlated values were obtained finding a value of 0.048 for *ϕ*_m_. This maximum volume packing fraction is similar to those values obtained by Fisa [74] and Utracki et al. [75] when studied flaky mica particles.

### Density

**Fig. 7:**
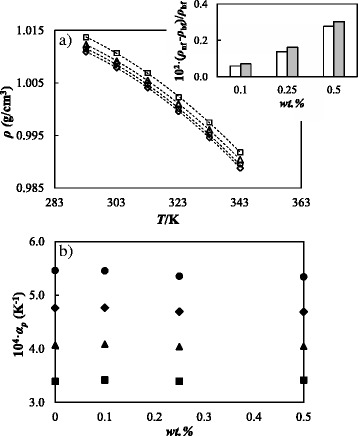
Volumetric behavior of GOnP/(EG + W) nanofluid set. **a** Temperature dependence of density, *ρ*, at different mass fractions: *diamond*, base fluid 0%; *circle*, 0.10%; *triangle*, 0.25%; and *square*, 0.50%. The *dashed lines* represent the fitted second-order polynomial, Eq. (6). The *inset* shows density enhancements of the nanofluids in relation to the base fluid, 10^2^ (*ρ*_nf_ − *ρ*_bf_)/*ρ*_bf_, at 303.15 K (*white bars*) and 343.15 K (*gray bars*) for the different concentrations. **b** Isobaric thermal expansivity, *α*_*p*_, vs. nanoparticle mass fraction, wt.%, at different temperatures: *square*, 303.15 K; *triangle*, 313.15 K; *diamond*, 323.15 K; and *circle*, 333.15 K

Density was studied for the base fluid and the three designed nanofluids at temperatures between 293.15 and 343.15 K. Experimental results are depicted in Fig. 7a. A comparison between the values here presented for the base fluid and previous density data [33, 60] shows AADs% less than 0.05%. As usually happens with nanofluids, density increases with nanoadditive concentration. These increases slightly rise with temperature ranging from 0.28 to 0.30% for the 0.50% mass concentration, as shown in the inset of the Fig. 7a. For each fluid, the temperature dependence of density was described by using a second-order polynomial:

6 $$ \rho (T)={b}_0+{b}_1\cdotp T+{b}_2\cdotp {T}^2 $$

where the *b*_i_ adjustable coefficients were obtained by using a least-square fitting and are gathered in Table 1. As shown in Fig. 7a, this equation provides a good density data description with standard deviations lower than 2 × 10^−4^ g cm^−3^. In addition, isobaric thermal expansivities, *α*_*p*_ = –(1/*ρ*) · (δ*ρ*/δ*T*)_*p*_, were numerically obtained through the derivatives of the polynomial fits of density. Figure 7b shows *α*_*p*_ values for the base fluid and the three studied nanofluids at different temperatures. As can be observed, this property slightly decreases with concentration, diminutions reaching up to 2% for 0.50% mass concentration at 333.15 K. This reduction in *α*_*p*_ with nanoparticle concentration agrees with previous nanofluid literature [76, 77].

### Nanofluid Comparison Based on Thermophysical Properties

As pointed out, different figures of merit based on the thermophysical properties can be used to compare the heat transfer performance or pumping power of nanofluids to those of the base fluid. In the case of fully developed internal laminar flow conditions, a merit criterion to assess the thermal capability is the ratio between viscosity and thermal conductivity enhancements [78–80]:

7 $$ \frac{C_{\eta }}{C_k}=\frac{{\left({\eta}_{\mathrm{nf}}-{\eta}_{\mathrm{bf}}\right)/\eta}_{\mathrm{bf}}}{\left({k}_{\mathrm{nf}}-{k}_{\mathrm{bf}}\right)/{k}_{\mathrm{bf}}}\le 4 $$

Thus, the increase in dynamic viscosity must be lower than four times the thermal conductivity improvement so that the replacement is beneficial in terms of thermal energy. Regarding the turbulent flow conditions, the heat transfer performance can be evaluated through the Mouromtseff number (Mo) [37] which is defined as:

8 $$ \mathrm{M}\mathrm{o}=\frac{\rho^{0.8}\cdot {k}^{0.67}\cdot {c}_p^{0.33}}{\eta^{0.47}},\kern6em \frac{{\mathrm{Mo}}_{\mathrm{nf}}}{{\mathrm{Mo}}_{\mathrm{bf}}}>1 $$

Higher Mo numbers indicate higher heat transfer capabilities and, consequently, a Mo_nf_/Mo_bf_ ratio higher than one is desirable.

In a circular tube with a uniform flux at the wall and considering that the mass flow rate of both base fluid and nanofluids is the same, the increment in pumping power can be assessed by using the following expressions for laminar and turbulent flow conditions, respectively [81]:

9 $$ \frac{{\overset{.}{W}}_{\mathrm{nf}}}{{\overset{.}{W}}_{\mathrm{bf}}}=\left(\frac{\eta_{\mathrm{nf}}}{\eta_{\mathrm{bf}}}\right)\cdot {\left(\frac{\rho_{\mathrm{bf}}}{\rho_{\mathrm{nf}}}\right)}^2 $$

10 $$ \frac{{\overset{.}{W}}_{\mathrm{nf}}}{{\overset{.}{W}}_{\mathrm{bf}}}={\left(\frac{\eta_{\mathrm{nf}}}{\eta_{\mathrm{bf}}}\right)}^{0.25}\cdot {\left(\frac{\rho_{\mathrm{bf}}}{\rho_{\mathrm{nf}}}\right)}^2 $$

Moderate Ẇ_nf_/Ẇ_bf_ ratios are needed to avoid increasing the energy consumption due to fluid pumping.

In order to perform these analyses, the experimental values of thermal conductivity, dynamic viscosity, and density were utilized, while specific heat capacities were obtained by using the following weighted average equation [82]:

11 $$ {c}_{p,\mathrm{n}\mathrm{f}}=\varphi \cdot {c}_{p,\mathrm{n}\mathrm{p}}+\left(1-\varphi \right)\cdot {c}_{p,\mathrm{b}\mathrm{f}} $$

**Fig. 8:**
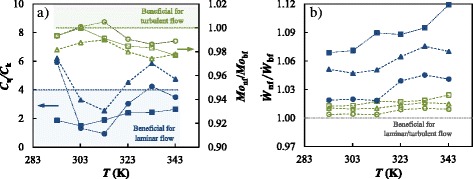
FoMs of GOnP/(EG + W) nanofluids based on thermophysical properties. **a** *C*_*η*_/*C*_*k*_ and Mo_nf_/Mo_bf_, and **b** ${\overset{.}{W}}_{\mathrm{nf}}/{\overset{.}{W}}_{\mathrm{bf}}$ for laminar (*filled symbols*) and turbulent flow (*empty symbols*) conditions; *circle*, 0.10%; *triangle*, 0.25%; and *square*, 0.50% mass concentrations

In order to carry out these calculations, the heat capacity data of graphene oxide and base fluid were obtained from literature [28, 33]. Figure 8 depicts the temperature dependence of these figures of merit for the different studied nanofluids. Under laminar flow rate conditions only the 0.50 wt.% GnOP concentration is within the benefit zone in the entire studied temperature range. However, for turbulent flow, this same concentration would obtain an improvement only at 303 K (Fig. 8a). In addition, pumping power rises with the concentration of graphene platelets up to 2.4 and 11.9% for laminar and turbulent flow conditions, respectively. Although pumping power increases with temperature under both flow regimens, increases are more appreciable under laminar conditions for which variations reach up to 5% throughout the analyzed range, as can be seen in Fig. 8b.

## Conclusions

New nanofluids consisting of GOnP dispersions in an (ethylene glycol + water) mixture were designed following a two-step process. According to zeta potential and dynamic light scattering analyses, dispersions exhibit zeta potentials of around 40 mV and trimodal apparent size distributions. A careful study of the thermodynamic profile of this nanofluid set shows that thermal conductivity, dynamic viscosity, and density increase with nanoplatelet mass concentration up to (5, 12.6, and 0.3)%, respectively. Temperature has a clear effect on the dynamic viscosity and density increases but not on the thermal conductivity enhancements. The temperature or nanoparticle behaviors of the two transport properties were also described by using Nan, Vogel-Fulcher-Tamman (VFT), or Maron and Pierce (MP) models with AADs% of (0.6, 1.6, and 1.3)%, respectively. Based on an analysis of different figures of merit and using the reported thermophysical property data of the nanofluids and base fluid, no significant enhancements in the heat transfer performance would be expected for the studied GOnP concentrations under turbulent conditions, while the pumping power increases would reach up to 2.4 and 11.9% for turbulent and laminar flow conditions, respectively.

## Abbreviations

AAD%: Average absolute deviation
αp: Isobaric thermal expansivity (K^−1^)
bf: Base fluid
*c*_*p*_: Specific heat capacity (J g^−1^ K^−1^)
EG: Ethylene glycol
GnP: Graphene nanoplatelet
GO: Graphene oxide
GOnP: Graphene oxide nanoplatelet
*k*: Thermal conductivity (W m^−1^ K^−1^)
*L*_ii_, *β*_ii_: Coefficients of Nan model
Mo: Mouromtseff number
nf: Nanofluid
np: Nanoparticle
*T*: Temperature (K)
Vol.%: Percentual volume concentration
wt.%: Percentual mass concentration
Ẇ: Pumping power (W)
W: Water
$$ \overset{.}{\gamma } $$: Shear rate (s^−1^)
*η*: Dynamic viscosity (mPa s)
*η*_*0*_, *D*, *T*_*0*_: Adjustable parameters of Vogel-Fulcher-Tamman (VFT) model
*ρ*: Density (g cm^−3^)
*τ*: Shear stress (Pa)
*φ*: Mass fraction
*ϕ*: Volume fraction
*ϕ*_a_, *ϕ*_m_: parameters of Maron-Pierce (MP) relationship

## References

[1] Mehrali M, Sadeghinezhad E, Rosen MA, Tahan Latibari S, Metselaar HSC, Kazi SN (2015). Effect of specific surface area on convective heat transfer of graphene nanoplatelet aqueous nanofluids. Exp Therm Fluid Sci.

[2] Żyła G (2016). Thermophysical properties of ethylene glycol based yttrium aluminum garnet (Y3Al5O12–EG) nanofluids. Int J Heat Mass Transf.

[3] Mehrali M, Sadeghinezhad E, Rosen MA, Akhiani AR, Tahan Latibari S, Metselaar HSC (2015). Heat transfer and entropy generation for laminar forced convection flow of graphene nanoplatelets nanofluids in a horizontal tube. Int Commun Heat Mass.

[4] Zyła G, Cholewa M, Witek A (2012). Dependence of viscosity of suspensions of ceramic nanopowders in ethyl alcohol on concentration and temperature. Nanoscale Res Lett.

[5] Park EJ, Park SD, Bang IC, Park YB, Park HW (2012). Critical heat flux characteristics of nanofluids based on exfoliated graphite nanoplatelets (xGnPs). Mater Lett.

[6] Lee C, Wei X, Kysar JW, Hone J (2008). Measurement of the elastic properties and intrinsic strength of monolayer graphene. Science.

[7] Sun B, Wang B, Su D, Xiao L, Ahn H, Wang G (2012). Graphene nanosheets as cathode catalysts for lithium-air batteries with an enhanced electrochemical performance. Carbon.

[8] Balandin AA, Ghosh S, Bao W, Calizo I, Teweldebrhan D, Miao F, Lau CN (2008). Superior thermal conductivity of single-layer graphene. Nano Lett.

[9] Novoselov KS, Geim AK, Morozov SV, Jiang D, Zhang Y, Dubonos SV, Grigorieva IV, Firsov AA (2004). Electric field in atomically thin carbon films. Science.

[10] Bianco A (2013). All in the graphene family—a recommended nomenclature for two-dimensional carbon materials. Carbon.

[11] Mohd Zubir MN, Badarudin A, Kazi SN, Huang NM, Misran M, Sadeghinezhad E, Mehrali M, Yusoff N (2015). Highly dispersed reduced graphene oxide and its hybrid complexes as effective additives for improving thermophysical property of heat transfer fluid. Int J Heat Mass Tran.

[12] Lee GJ, Rhee CK (2014). Enhanced thermal conductivity of nanofluids containing graphene nanoplatelets prepared by ultrasound irradiation. J Mater Sci.

[13] Yarmand H (2015). Graphene nanoplatelets-silver hybrid nanofluids for enhanced heat transfer. Energy Convers Manage.

[14] Azizi M, Hosseini M, Zafarnak S, Shanbedi M, Amiri A (2013). Experimental analysis of thermal performance in a two-phase closed thermosiphon using graphene/water nanofluid. Ind Eng Chem Res.

[15] Kole M, Dey TK (2013). Investigation of thermal conductivity, viscosity, and electrical conductivity of graphene based nanofluids. J Appl Phys.

[16] Amiri A, Sadri R, Shanbedi M, Ahmadi G, Chew BT, Kazi SN, Dahari M (2015). Performance dependence of thermosyphon on the functionalization approaches: an experimental study on thermo-physical properties of graphene nanoplatelet-based water nanofluids. Energy Convers Manage.

[17] Kazi SN, Badarudin A, Zubir MNM, Ming HN, Misran M, Sadeghinezhad E, Mehrali M, Syuhada NI (2015). Investigation on the use of graphene oxide as novel surfactant to stabilize weakly charged graphene nanoplatelets. Nanoscale Res Lett.

[18] Li X, Chen Y, Mo S, Jia L, Shao X (2014). Effect of surface modification on the stability and thermal conductivity of water-based SiO_2_-coated graphene nanofluid. Thermochim Acta.

[19] Sadeghinezhad E, Mehrali M, Saidur R, Tahan Latibari S, Akhiani AR, Metselaar HSC (2016). A comprehensive review on graphene nanofluids: recent research, development and applications. Energy Convers Manage.

[20] Yu W, Xie H, Wang X (2011). Significant thermal conductivity enhancement for nanofluids containing graphene nanosheets. Physics Letters A.

[21] Ahammed N, Asirvatham LG, Wongwises S (2015). Effect of volume concentration and temperature on viscosity and surface tension of graphene–water nanofluid for heat transfer applications. J Therm Anal Calorim.

[22] Sarsam WS, Amiri A, Kazi SN, Badarudin A (2016). Stability and thermophysical properties of non-covalently functionalized graphene nanoplatelets nanofluids. Energy Convers Manage.

[23] Akhavan-Zanjani H, Saffar-Avval M, Mansourkiaei M, Ahadi M, Sharif F (2014). Turbulent convective heat transfer and pressure drop of graphene-water nanofluid flowing inside a horizontal circular tube. J Disper Sci Technol.

[24] Sen Gupta S, Manoj Siva V, Krishnan S, Sreeprasad TS, Singh PK, Pradeep T, Das SK (2011). Thermal conductivity enhancement of nanofluids containing graphene nanosheets. J Appl Phys.

[25] Baby TT, Ramaprabhu S (2010). Investigation of thermal and electrical conductivity of graphene based nanofluids. J Appl Phys.

[26] Yu W, Xie H, Chen W (2010). Experimental investigation on thermal conductivity of nanofluids containing graphene oxide nanosheets. J Appl Phys.

[27] Paredes JI, Villar-Rodil S, Martínez-Alonso A, Tascón JMD (2008). Graphene oxide dispersions in organic solvents. Langmuir.

[28] Pop E, Varshney V, Roy AK (2012). Thermal properties of graphene: fundamentals and applications. MRS Bull.

[29] Teng CC, Ma CCM, Lu CH, Yang SY, Lee SH, Hsiao MC, Yen MY, Chiou KC, Lee TM (2011). Thermal conductivity and structure of non-covalent functionalized graphene/epoxy composites. Carbon.

[30] Rasheed AK, Khalid M, Rashmi W, Gupta TCSM, Chan A (2016). Graphene based nanofluids and nanolubricants—review of recent developments. Renew Sust Energ Rev.

[31] Hadadian M, Goharshadi EK, Youssefi A (2014). Electrical conductivity, thermal conductivity, and rheological properties of graphene oxide-based nanofluids. J Nanopart Res.

[32] Dimiev AM, Alemany LB, Tour JM (2013). Graphene oxide. Origin of acidity, its instability in water, and a new dynamic structural model. ACS Nano.

[33] Melinder A (2010). Properties of secondary working fluids for indirect systems.

[34] Azmi WH, Sharma KV, Mamat R, Najafi G, Mohamad MS (2016). The enhancement of effective thermal conductivity and effective dynamic viscosity of nanofluids—a review. Renew Sust Energ Rev.

[35] Żyła G, Fal J, Traciak J, Gizowska M, Perkowski K (2016). Huge thermal conductivity enhancement in boron nitride—ethylene glycol nanofluids. Mater Chem Phys.

[36] Hermida-Merino C, Pérez-Rodriguez M, Piñeiro MM, Pastoriza-Gallego MJ (2016). Evidence of viscoplastic behavior of exfoliated graphite nanofluids. Soft Matter.

[37] Mouromtseff IE (1942). Water and Forced-Air Cooling of Vacuum Tubes Nonelectronic Problems in Electronic Tubes. Proceedings of the Institute of Electrical and Electronics Engineers (IEEE), New Yersey, pp 190–205

[38] Lemmon EW, Huber ML, McLinden MO (2010). NIST Standard Reference Database 23. Reference Fluid Thermodynamic and Transport Properties (REFPROP), National Institute of Standards and Technology.

[39] Baby TT, Ramaprabhu S (2011). Enhanced convective heat transfer using graphene dispersed nanofluids. Nanoscale Res Lett.

[40] Jyothirmayee Aravind SS, Ramaprabhu S (2011). Surfactant free graphene nanosheets based nanofluids by in-situ reduction of alkaline graphite oxide suspensions. J Appl Phys.

[41] Hajjar Z, Rashidi AM, Ghozatloo A (2014). Enhanced thermal conductivities of graphene oxide nanofluids. Int Commun Heat Mass.

[42] Amiri A, Sadri R, Shanbedi M, Ahmadi G, Kazi SN, Chew BT, Zubir MNM (2015). Synthesis of ethylene glycol-treated graphene nanoplatelets with one-pot, microwave-assisted functionalization for use as a high performance engine coolant. Energy Convers Manage.

[43] Ijam A, Saidur R, Ganesan P, Moradi GA (2015). Stability, thermo-physical properties, and electrical conductivity of graphene oxide-deionized water/ethylene glycol based nanofluid. Int J Heat Mass Tran.

[44] Geng Y, Wang SJ, Kim J-K (2009). Preparation of graphite nanoplatelets and graphene sheets. J Colloid Interface Sci.

[45] Agromayor R, Cabaleiro D, Pardinas AA, Vallejo JP, Fernandez-Seara J, Lugo L (2016). Heat transfer performance of functionalized graphene nanoplatelet aqueous nanofluids. Mater.

[46] Fedele L, Colla L, Bobbo S, Barison S, Agresti F (2011). Experimental stability analysis of different water-based nanofluids. Nanoscale Res Lett.

[47] Dhar P, Sen Gupta S, Chakraborty S, Pattamatta A, Das SK (2013). The role of percolation and sheet dynamics during heat conduction in poly-dispersed graphene nanofluids. Appl Phys Lett.

[48] Fedele L, Colla L, Bobbo S (2012). Viscosity and thermal conductivity measurements of water-based nanofluids containing titanium oxide nanoparticles. Int J Refrig.

[49] Bobbo S, Fedele L, Benetti A, Colla L, Fabrizio M, Pagura C, Barison S (2012). Viscosity of water based SWCNH and TiO_2_ nanofluids. Exp Therm Fluid Sci.

[50] Cabaleiro D, Pastoriza-Gallego MJ, Piñeiro MM, Legido JL, Lugo L (2012). Thermophysical properties of (diphenyl ether + biphenyl) mixtures for their use as heat transfer fluids. J Chem Thermodyn.

[51] Sun T, Teja AS (2003). Density, viscosity, and thermal conductivity of aqueous ethylene, diethylene, and triethylene glycol mixtures between 290 K and 450 K. J Chem Eng Data.

[52] Bohne D, Fischer S, Obermeier E (1984). Thermal conductivity, density, viscosity, and Prandt-numbers of ethylene glycol-water mixtures. Ber Bunsenges Phys Chem.

[53] Yu W, Xie H, Bao D (2010). Enhanced thermal conductivities of nanofluids containing graphene oxide nanosheets. Nanotechnology.

[54] Maxwell JC (1882). A treatise on electricity and magnetism.

[55] Nan CW, Birringer R, Clarke DR, Gleiter H (1997). Effective thermal conductivity of particulate composites with interfacial thermal resistance. J Appl Phys.

[56] Buongiorno J (2009). A benchmark study on the thermal conductivity of nanofluids. J Appl Phys.

[57] Kamatchi R, Venkatachalapathy S, Abhinaya SB (2015). Synthesis, stability, transport properties, and surface wettability of reduced graphene oxide/water nanofluids. Int J Therm Sci.

[58] Mehrali M, Sadeghinezhad E, Latibari S, Kazi S, Mehrali M, Zubir MNBM, Metselaar HS (2014). Investigation of thermal conductivity and rheological properties of nanofluids containing graphene nanoplatelets. Nanoscale Res Lett.

[59] Nika DL, Pokatilov EP, Askerov AS, Balandin AA (2009). Phonon thermal conduction in graphene: role of Umklapp and edge roughness scattering. Phys Rev B Condens Matter Mater Phys.

[60] Tsierkezos NG, Molinou IE (1998). Thermodynamic properties of water + ethylene glycol at 283.15, 293.15, 303.15, and 313.15 K. J Chem Eng Data.

[61] Yang C, Ma P, Jing F, Tang D (2003). Excess molar volumes, viscosities, and heat capacities for the mixtures of ethylene glycol + water from 273.15 K to 353.15 K. J Chem Eng Data.

[62] Iulian O, Ciocîrlan O (2010). Viscosity and density of systems with water, 1,4-dioxane and ethylene glycol between (293.15 and 313.15) K. I. binary systems. Rev. Roumaine Chim.

[63] Ma W, Yang F, Shi J, Wang F, Zhang Z, Wang S (2013). Silicone based nanofluids containing functionalized graphene nanosheets. Colloids Surf A Physicochem Eng Asp.

[64] Cabaleiro D, Segovia JJ, Martín MC, Lugo L (2016). Isobaric heat capacity at high pressure, density, and viscosity of (diphenyl ether + biphenyl) mixtures. J Chem Thermodyn.

[65] Hodge IM (1996). Strong and fragile liquids—a brief critique. J Non Cryst Solids.

[66] Einstein A (1906). A new determination of molecular dimensions. Ann Phys.

[67] Maron SH, Pierce PE (1956). Application of ree-eyring generalized flow theory to suspensions of spherical particles. J Colloid Sci.

[68] Krieger IM, Dougherty TJ (1959). A mechanism for non-Newtonian flow in suspensions of rigid spheres. J Rheol.

[69] Estellé P, Halelfadl S, Maré T (2014). Lignin as dispersant for water-based carbon nanotubes nanofluids: impact on viscosity and thermal conductivity. Int Commun Heat Mass Transf.

[70] Mueller S, Llewellin EW, Mader HM (2010). The rheology of suspensions of solid particles. Proc R Soc A Math Phys Eng Sci.

[71] Halelfadl S, Estellé P, Aladag B, Doner N, Maré T (2013). Viscosity of carbon nanotubes water-based nanofluids: influence of concentration and temperature. Int J Therm Sci.

[72] Estellé P (2016). Comment on “Performance of CNT-water nanofluid as coolant fluid in shell and tube intercooler of a LPG absorber tower”. Int J Heat Mass Transf.

[73] King JA, Via MD, Morrison FA, Wiese KR, Beach EA, Cieslinski MJ, Bogucki GR (2012). Characterization of exfoliated graphite nanoplatelets/polycarbonate composites: Electrical and thermal conductivity, and tensile, flexural, and rheological properties. J Compos Mater.

[74] Fisa B, Vu-Khanh T, Remillard B (1988). Extrusion of mica filled polypropylene. J Thermoplast Compos Mater.

[75] Utracki LA, Favis BD, Fisa B (1984). Dynamic and steady state flow of polypropylene/mica systems. Polym Compos.

[76] Cabaleiro D, Pastoriza-Gallego MJ, Gracia-Fernández C, Piñeiro MM, Lugo L (2013). Rheological and volumetric properties of TiO_2_- ethylene glycol nanofluids. Nanoscale Res Lett.

[77] Cabaleiro D, Pastoriza-Gallego MJ, Piñeiro MM, Lugo L (2013). Characterization and measurements of thermal conductivity, density and rheological properties of zinc oxide nanoparticles dispersed in (ethane-1,2-diol + water) mixture. J Chem Thermodyn.

[78] Prasher R, Song D, Wang J, Phelan P (2006). Measurements of nanofluid viscosity and its implications for thermal applications. Appl Phys Lett.

[79] Halelfadl S, Maré T, Estellé P (2014). Efficiency of carbon nanotubes water based nanofluids as coolants. Exp Therm Fluid Sci.

[80] Zin V, Barison S, Agresti F, Colla L, Pagura C, Fabrizio M (2016). Improved tribological and thermal properties of lubricants by graphene based nano-additives. RSC Adv.

[81] Mansour RB, Galanis N, Nguyen CT (2007). Effect of uncertainties in physical properties on forced convection heat transfer with nanofluids. Appl Therm Eng.

[82] Cabaleiro D, Gracia-Fernández C, Legido JL, Lugo L (2015). Specific heat of metal oxide nanofluids at high concentrations for heat transfer. Int J Heat Mass Tran.

